# Anti-Xa Monitoring of Apixaban (ZyQuis) in Venous Thrombo-Embolism and Atrial Fibrillation

**DOI:** 10.1177/10760296241249167

**Published:** 2024-04-24

**Authors:** Elise Schapkaitz, Byron Ter Morshuizen, Melanie Mc Cree, Barry F. Jacobson

**Affiliations:** 1 Department of Molecular Medicine and Haematology, 37708 School of Pathology, Faculty of Health Sciences, University of the Witwatersrand, Johannesburg, South Africa; 2 South African Society of Thrombosis and Haemostasis, Johannesburg, South Africa

**Keywords:** Anti-Xa, apixaban, venous thrombo-embolism, atrial fibrillation, generic

## Abstract

Apixaban is a direct oral Xa inhibitor and is indicated for the treatment of venous thrombo-embolism (VTE) and prevention of stroke in atrial fibrillation (AF). Recently, a generic (ZyQuis, Zydus Lifesciences Limited, India) has received Food and Drug Administration approval. While bioequivalence has been demonstrated with Eliquis (Bristol-Myers Squibb/Pfizer, UK), it is necessary to monitor its effectiveness prior to acceptance in medical practice. This prospective study independently evaluated Apixaban (ZyQuis) at two accredited laboratories. Participants were converted from Warfarin or Rivaroxaban to Apixaban 5 mg bd for a duration of one month. Peak anti-Xa levels were measured 3-4 h post the morning dose. The samples were processed on the Atellica COAG 360 (Siemens Healthineers, Marburg, Germany) analyzers with a chromogenic anti-Xa assay (Innovance, reference interval 69-321 ng/mL). There were 26 participants; 5 men, 21 women; mean ± standard deviation age of 46 ± 12 years. Indications for anticoagulation included: VTE (88.5%) and AF (11.5%). 69.2% of the participants had at least one comorbidity. 96.2% of the anti-Xa levels were within the laboratory's 95% reference interval. Mean anti-Xa activity was 191 ± 69 ng/mL and 186 ± 68 ng/mL measured at respective laboratories. Mean differences in anti-Xa measurements represented by Bland–Altman statistics were small (bias of −2.6%, 95% confidence interval −1.11 to −4.09) and a strong correlation was observed on Deming regression analysis (0.995). Apixaban (ZyQuis) was effective for the management of VTE and AF as evidenced by anti-Xa activity.

## Background

Vitamin K antagonists (VKA) such as Warfarin are highly effective treatments for venous thrombo-embolism (VTE) and preventing stroke in patients with non-valvular atrial fibrillation (AF). While VKA represent a cost-effective alternative for resource-limited countries, the use of VKA is limited by its narrow target range, drug and dietary interactions, regular monitoring requirements, and the risk of bleeding. More recently, the direct oral anticoagulants (DOAC) have been shown to be equivalent or superior to Warfarin in preventing stroke and treating VTE.^1-5^ A meta-analysis of the DOAC trials compared to VKA showed reduced rates of mortality, stroke or systemic embolism, and hemorrhagic stroke.^6^ Current guidelines suggest DOAC use over Warfarin in both non-valvular AF and non-cancer VTE patients.^7,8^

DOAC include the oral direct thrombin inhibitor Dabigatran and direct factor Xa inhibitors including, Apixaban, Edoxaban, and Rivaroxaban. Apixaban offers several advantages as compared to other available DOAC. Apixaban is less dependent on kidney function for clearance and is thus, the preferred DOAC for patients with chronic kidney disease.^9^ Apixaban is associated with a lower risk of major bleeding. In a subgroup analysis of the ARISTOPHANES observational AF study, Apixaban was associated with a lower risk of major bleeding (hazards ratio 0.59, 95% CI 0.56-0.63), as compared to Warfarin as well as Rivaroxaban.^10^ Apixaban has rapid absorption and a 12-h half-life. The recommended dose of Apixaban is 5 mg twice daily. In patients with two or more of the following: age ≥80 years, body weight ≤60 kg, or serum creatinine level ≥ 133 micromol/L, the dose of Apixaban is 2.5 mg twice daily.

The price of DOAC is a major obstacle for healthcare systems. Expanded use of generic drugs represents an important alternative to ensure access to affordable and essential medicines. Nonetheless, while generic DOAC could help increase the proportion of patients who have access to optimal anticoagulation, bioequivalence is a concern for anticoagulants owing to the risks of bleeding and thrombotic outcomes as well as the relatively narrow therapeutic indices. In the case of Warfarin, earlier clinical studies revealed differences in the international normalised ratio (INR) levels of originator and generic Warfarin owing to the narrow therapeutic index of Warfarin.^11^

Recently a generic equivalent of Apixaban (ZyQuis, Zydus Lifesciences Limited, India) has received Food and Drug Administration approval. An open-label, randomized, two-period, two-treatment, two-sequence, crossover, balanced, single-dose oral bioequivalence study was performed by the manufacturer in healthy, adult, human subjects under fasting conditions. The oral bioavailability of test formulation Apixaban 5 mg was compared with the reference formulation, Eliquis (Apixaban) 5 mg (Bristol-Myers Squibb/Pfizer, UK) in 27 subjects. Blood samples collected at intervals over 48 h after administration of each dose showed bioequivalence. The 90% confidential interval of the geometric mean of the test to reference formulation for maximum observed concentration (Cmax) and area under the plasma concentration–time curve (AUC) were within 80% to 125%. Further, there was no serious adverse events reported in the study.^12^ While bioequivalence has been demonstrated between the generic equivalent and the reference formulation, Eliquis (Bristol-Myers Squibb/Pfizer, UK), it is, however, necessary to monitor its effectiveness prior to acceptance in medical practice. A prospective study was performed to independently evaluate Apixaban (ZyQuis) as determined by laboratory monitoring.

## Methods

### Study Design and Population

This study was performed at the Anticoagulation Clinic at the Charlotte Maxeke Johannesburg Academic Hospital, Johannesburg, South Africa between May and October 2023. The Anticoagulation Clinic consists of approximately 400 patients who are prescribed Rivaroxaban or Warfarin. The frequency of clinic visits for patients receiving Warfarin ranges from 1 to 4 weeks. Patients receiving Rivaroxaban are seen six-monthly. Consecutive patients ≥18 years receiving anticoagulation were invited to participate in the study. Indications included deep vein thrombosis diagnosed by compression ultrasound and/or pulmonary embolism diagnosed by computed tomography pulmonary angiogram or non-valvular AF as documented by electrocardiography. The following exclusion criteria were applied: age ≥ 80 years, body weight ≤ 60 kg, mechanical or bio-prosthetic heart valve replacement, rheumatic mitral stenosis—moderate or severe (mitral valve area ≤1.5 cm^2^), renal impairment (estimated glomerular filtration rate < 30 mL/min or Stage 4 or 5 chronic kidney disease), liver impairment (alanine aminotransferase/aspartate aminotransferase > 1.5 × upper limit of normal or Child-Pugh B or C), anti-phospholipid syndrome, stroke within the previous 7 day, drug interactions with DOAC agents (eg, those receiving P-glycoprotein drug efflux pump inducers, which can decrease the anticoagulant effect of DOAC and chronic antiviral agents, which may increase the anticoagulant effect of DOAC), concomitant Aspirin and Clopidogrel, known hypersensitivity to apixaban, pregnancy and/or breastfeeding, and non-adherence to twice-daily medication.

### Study Protocol

Participants were initiated on, or converted from Warfarin (Aspen Pharmacare Holdings Ltd, South Africa) or Rivaroxaban (IXarola, Bayer (Pty) Ltd, South Africa), to a fixed dose of Apixaban (ZyQuis) 5 mg bd for a duration of 1-month. The study included anti-Xa monitoring after 1 week and an assessment after 1 month of clinical outcomes and adverse events. Participants were supplied with a 24-h emergency contact number. Written informed consent to collect and use of anonymous data were obtained. The study was approved by the Human Research Ethics Committee of the University of the Witwatersrand (M230105).

### Data Collection

The following history was collected from patient records and interview: demographics; HIV, diagnosis, antiretroviral therapy and adherence; indication for anticoagulation therapy and duration; concomitant medications; time in therapeutic range on warfarin^13^; RIETE prediction score for bleeding; and previous thrombo-embolic complications on anticoagulation and lifestyle affecting adherence to Warfarin. The following clinical examinations were collected: resting blood pressure of the left arm, heart rate, and body mass index (BMI).

At the first visit, Apixaban 5 mg bd × 28 days was dispensed. For participants on Warfarin, Apixaban was started when the INR <2. For patients on Rivaroxaban, Apixaban was started 24 h after the last dose. After 1-week, blood was collected for anti-Xa monitoring. After 1-month, when participants discontinued the study drug, guidance was provided in making the transition to Warfarin or Rivaroxaban. Apixaban was overlapped with Warfarin until the INR ≥2. The following outcomes were recorded at the two study visits: treatment-emergent adverse events, recurrent thrombosis, systemic embolism, and bleeding. Bleeding was defined according to the criteria of the International Society on Thrombosis and Haemostasis (ISTH).^14^

### Laboratory Investigations

Venous blood for peak anti-Xa measurement was collected in 3.2% sodium citrate (Becton-Dickinson, Oxford, UK) 3-4 h post the morning dose after 1-week. The samples were centrifuged at 3500 × g for 15 min. The platelet-poor plasma was separated, frozen at −70°C, and transported to accredited laboratories (Ampath and Lancet Laboratories). The samples were processed on the Atellica COAG 360 (Siemens Healthineers, Marburg, Germany) analyzers with chromogenic Apixaban anti-Xa reagants (Innovance, reference interval 69-321 ng/mL). The manufacturer's % coefficient of variation is 4.05%.

### Data Analysis

Statistical analysis was performed with EP-evaluator Software (Colchester, VT, USA) using the Bland–Altman method and Deming regression statistical methods. Descriptive analysis of clinical and laboratory data was performed using Statistica^®^ version 13.2 TIBCO software (Palo Alto, California, USA). Normally distributed continuous data was presented as mean ± standard deviation (SD) and variables with non-Gaussian distribution as median (interquartile range [IQR]).

## Results

**Figure 1.**
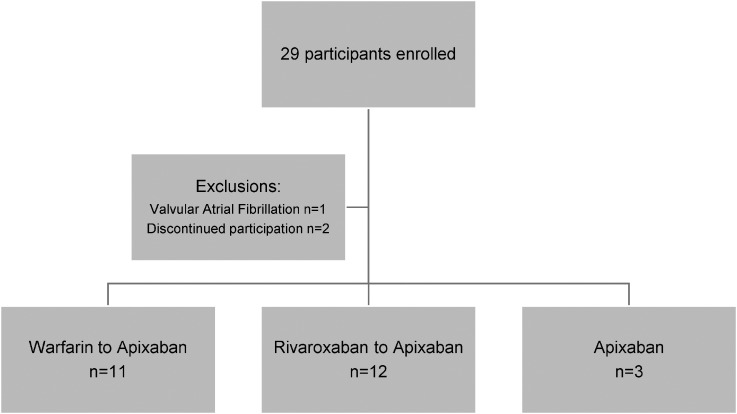
Flow of study patients.

A total of 29 consecutive adult participants with VTE or AF consented to participate in the study; one with valvular AF was excluded (Figure 1). A further two participants withdrew from the study as a result of grade one drug-related adverse events namely chest pain and headache. The baseline characteristics of the 26 patients who participated in the study are summarized in Table 1. The participants were predominantly of African ethnicity (84.6%); 5 men and 21 women; mean ± SD age of 46 ± 12 years. Indications for anticoagulation included: VTE (88.5%) and AF (11.5%). 69.2% of the participants had at least one comorbidity. The mean BMI was 33 ± 1.5 kg/m^2^ with 8.3% with BMI ≥ 40 kg/m^2^. According to the RIETE score, 80% were classified as low risk and 20% as intermediate risk for bleeding.

**Table 1.** Baseline Characteristics.

Characteristics	
Number	26
Demographics	
Age (years), mean ± SD	45.8 ± 12.3
Gender (n, %)	
Male	5 (80.8)
Female	21 (19.2)
Ethnicity (n, %)	
African	22 (84.6)
Caucasian	3 (11.5)
Other	1 (3.8)
Clinical characteristics	
BMI (kg/m^2^), median [IQR]	31.8 [9.1]
Comorbidities (n, %)^a^	
0	8 (30.8)
1	11 (42.3)
2	3 (11.5)
3	4 (15.4)
Vital signs, mean ± SD	
Heart rate (bpm)	75.3 ± 11.5
SBP (mm Hg)	130.9 ± 16.3
DBP (mm Hg)	89.2 ± 11.0
Laboratory investigations	
Creatinine (umol/L), mean ± SD (ref: 64-104)	75.2 ± 16.0
TTR on warfarin (%), median [IQR]	77 [50-100]

SD, standard deviation; IQR, interquartile range; BMI, body mass index; SBP, systolic blood pressure; DBP, diastolic blood pressure; TTR, time in therapeutic range

^a^Human immunodeficiency virus (n=8), hypertension (n=7), diabetes (n=3), cardiac failure (n=1), tuberculosis (n=1), peripheral vascular disease (n=1), previous stroke, transient ischemic attack, or embolism (n=2).

**Figure 2.**
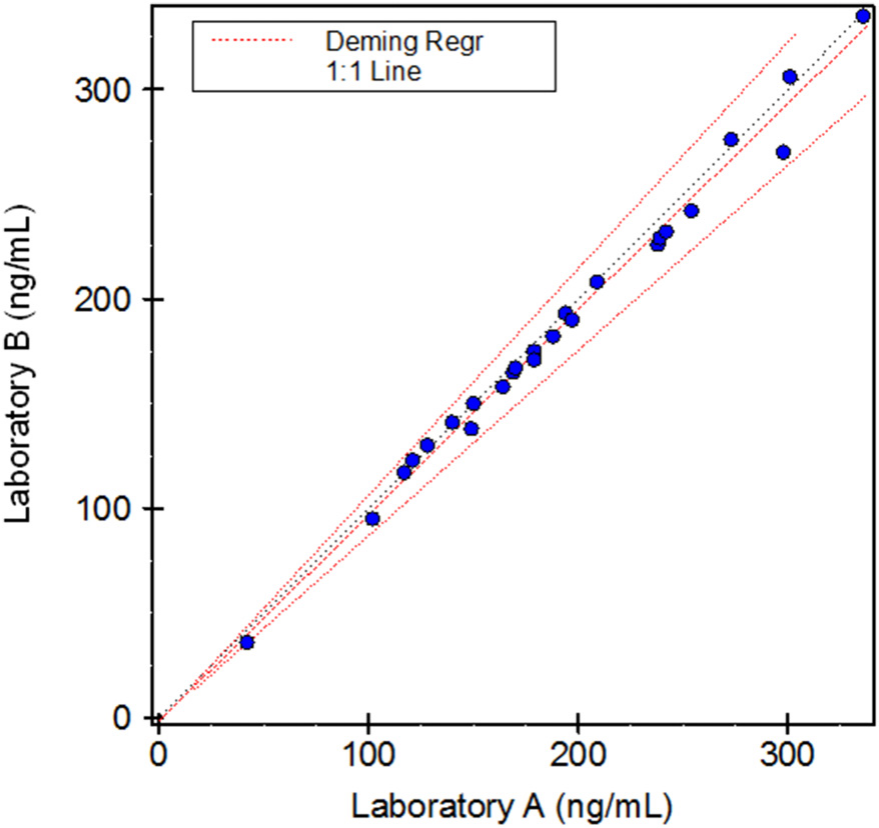
Deming regression analysis of anti-Xa activity at Lancet laboratory (A) and Ampath laboratory (B).

**Figure 3.**
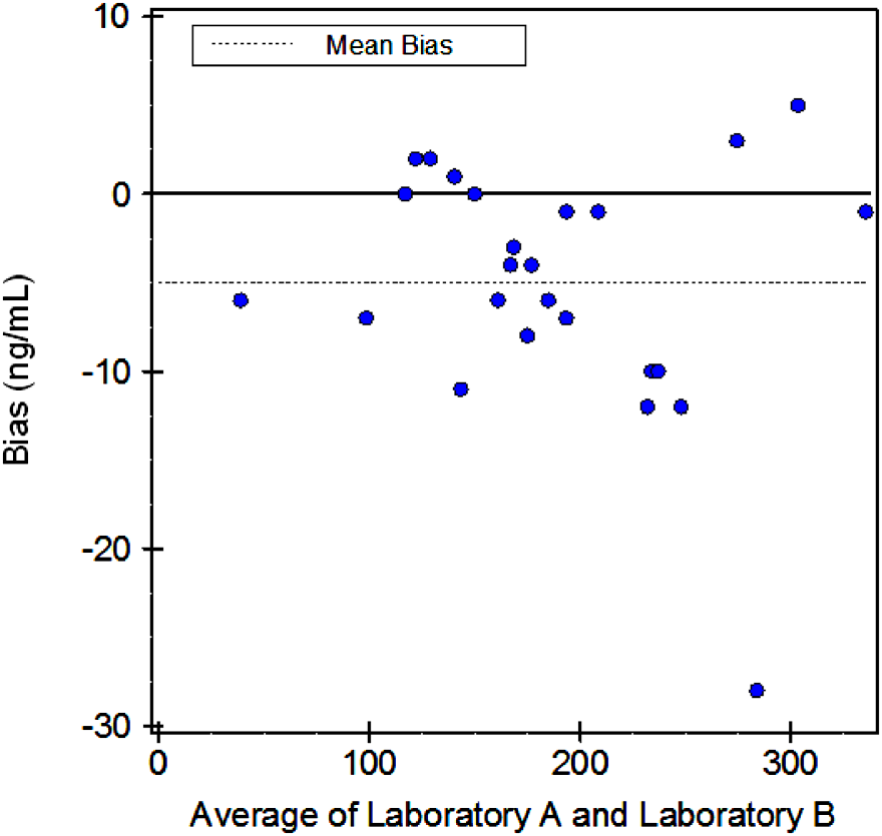
Bland–Altman statistics of anti-Xa activity at Lancet laboratory (A) and Ampath laboratory (B).

Of the anti-Xa levels measured, 96.2% were within the laboratory's 95% reference interval. Mean anti-Xa activity was 191 ± 69 ng/mL measured at Lancet Laboratory (A) and 186 ± 68 ng/mL measured at Ampath Laboratory (B). A strong correlation in anti-Xa measurements was observed on Deming regression analysis (0.995) (Figure 2) and mean differences in anti-Xa measurements represented by Bland–Altman statistics were small (bias of −2.6%, 95% confidence interval −1.11 to −4.09; Figure 3). There were no bleeding events or serious adverse events. After transitioning back to Warfarin, one patient developed a recurrent deep vein thrombosis.

## Discussion

In South Africa, the National Medicines Policy was introduced in 1996. Since then generics have been recommended for wider use in both the public and private sectors as a cost-saving measure once the manufacturer's market exclusivity period ends. For the generic Apixaban (ZyQuis) bioequivalence with Eliquis has been established previously as per regulatory guidelines. This study further evaluated Apixaban (ZyQuis) by laboratory monitoring. The findings suggest that appropriate substitution with Apixaban (ZyQuis) could substantially decrease costs for VTE treatment and stroke prevention in AF. Nonetheless, additional data about the effectiveness, safety, and costs associated with this treatment option are necessary to inform policy decisions.

In this study, the study participants were predominantly of African ethnicity, which is representative of the patient population, and of whom the majority had at least one co-morbidity. Apixaban (ZyQuis) was effective for the management of VTE and AF as evidenced by anti-Xa activity. Anti-Xa assays are regarded as the gold standard for the measurement of therapeutic DOAC levels and previous studies of Eliquis (Apixaban) have shown that anti-Xa measurements display a linear dose–response to Apixaban.^15^ Recently, clinical evidence has emerged regarding the efficacy and safety of DOAC in patients with extreme obesity.^16^ The updated guidance statement from the ISTH suggests that standard doses of Apixaban are appropriate for patients with high BMI and weight.^17,18^ Participants with a BMI ≥ 40 kg/m^2^ accounted for a small proportion of the current study, nonetheless, anti-Xa levels were within the laboratory's 95% reference interval. In South Africa, a resource-limited country, there is a bimodal distribution of BMI, with a rise in obesity particularly in urban settings. Peak or trough anti-Xa monitoring remains a matter of debate for patients with high BMI and weight owing to insufficient data with regard to safety and efficacy outcomes.

In the follow-up period of 1 month, there were no bleeding or adverse events. The majority of the study participants were classified as low-risk according to the RIETE score. After transitioning back to warfarin, one patient developed a recurrent deep vein thrombosis and close monitoring is advised during such switch periods until a therapeutic INR is achieved.

The study findings should be interpreted in light of the following limitations. Firstly, the sample size was small. Secondly, participants with AF were underrepresented in the study. Thirdly, anti-Xa measurements were performed using the same analysis and reagents at the respective laboratories. Lastly, participants with potential drug interactions with Apixaban (ZyQuis) were excluded from this study. Apixaban interacts significantly with P-glycoprotein and cytochrome P450 inducers and inhibitors.^19^ Apixaban is 87% protein bound and interactions with drugs with a higher protein affinity can also result in a possible increase of drug levels. Additional clinical pharmacokinetic studies are required to determine the drug-to-drug interactions with Apixaban (ZyQuis) in order to advise possible strategies, including dosage reductions and anti-Xa laboratory monitoring.

In conclusion, the results of this study suggest that substitution with Apixaban (ZyQuis) may promote considerable savings in health care costs without affecting the quality or the therapeutic effect—as evidenced by anti-Xa activity. Close monitoring is advised during any switch periods. Future studies are required to explore real-world evidence to promote the appropriate use of generics such as Apixaban (ZyQuis) for the management of VTE and AF and to provide treatment guidance for implementation across different populations and healthcare settings.
